# What nutrition advice is freely available for gymnasts, coaches and parents on their member National Governing Body webpages?

**DOI:** 10.1177/02601060241274440

**Published:** 2024-08-28

**Authors:** Jessica Riman, Karen M Keane, Kirsty M Hicks, Georgia Allen, Daniel J Peart

**Affiliations:** 1 Department of Sport, Exercise and Rehabilitation, 5995 Northumbria University, Newcastle-Upon-Tyne, UK; 2 Department of Sport, Exercise & Nutrition, 8816 Galway-Mayo Institute of Technology, Galway, Ireland

**Keywords:** Nutrition information, nutrition advice, gymnastics, coaches, parents, nutrition resources

## Abstract

The purpose of this content analysis was to ascertain what nutrition advice or information is freely available on a global scale through each National Governing Body (NGB) webpage. In total, 52 NGBs and the International Federation of Gymnastics (FIG) were identified based on the FIG November 2022 world rankings for both male and female athletes in all disciplines. Concluding observations were that publicly available nutrition advice is limited across the global gymnastics platforms. Conflicting advice was also identified within some organisations surrounding the weighing of gymnasts for hydration purposes and some macronutrient discrepancies thus, potentially impacting the clarity of the message for the reader. Based on this content analysis recommendations for future practice include providing clarity and provision of tools to measure hydration, dietary recommendations should be clear and specific, and a more centralised approach to standardise advice and publicly available information.

## Introduction

Gymnastics is a highly skilled aesthetic sport involving a large training and competition load (Dallas et al., 2017; Kaur and Koley, 2019; Sweeney et al., 2018). As a result of these high demands, appropriate nutrient intake for training, competition and recovery is paramount to ensuring successful athletic performance and maintaining good health (Dallas et al., 2017). Although it is generally accepted that athletes need appropriate fuelling, there is a question as to whether gymnasts or their support personnel are aware of recommended energy requirements and how or where they should seek information to support this (Dallas et al., 2017; Juzwiak and Ancona-Lopez, 2004). Many factors contribute to the importance of meeting energy requirements in gymnasts such as energy expenditure, training load and the relative age of the gymnasts (Fogelholm et al., 1995; Goulart et al., 2022; Kawano et al., 2002; Purcell, 2013). However, consistently in the literature gymnasts display lower energy intakes than reported energy expenditures (Fogelholm et al., 1995; Kawano et al., 2002), implying an energy deficit of between 300 and 700 kcals which can have a detrimental effect on performance (Cupisti et al., 2000; Gifari et al., 2020; Matsushita et al., 2019). One of the potential barriers for gymnasts consuming optimal intakes could be the influence of key athletic support personnel. Coaches spend a large portion of time with their athletes, particularly at the elite level, and are responsible for the gymnast, their training and squad selection (Torres-McGehee et al., 2012). It is therefore unsurprising that coaches naturally become a key contact point for athletes to seek information and support (Slater et al., 2003). Additionally, as gymnasts begin intensive training at a young age, their parents will likely have primary responsibility for their dietary intake (Gjaka et al., 2021). Parents provide an environment in which children relate to food (Scaglioni et al., 2008). However, it has been reported in the literature that parents would like to enhance their nutritional and well-being knowledge, which suggests that they might feel ill-equipped to support their children with their sport specific needs (Gjaka et al., 2021; Thomas et al., 2012).

If an athlete, coach, parent or guardian were to look for nutrition information, a logical place to look in the first instance would be the member National Governing Body (NGB) webpage. Therefore, it is important to review what information is offered by gymnastic NGBs to understand whether the information is aligned with the literature, and what each NGB considers important for their athletes to optimise health and performance. The purpose of this analysis was to ascertain what nutrition advice is freely available on a global platform for gymnasts.

## Methods

A content analysis was used to extract the data (Harwood and Garry, 2003; Kleinheksel et al., 2020). In the first instance, the International Federation of Gymnastics (FIG) world rankings were consulted including all Olympic disciplines, trampoline, artistic and rhythmic gymnastics disciplines for both male and female gymnasts. A list was created of all the world ranked gymnasts, and a search strategy was developed by identifying the NGBs where the gymnast was involved. National Governing Body webpages were located either by searching manually or looking through the FIG webpages. When a webpage was found, it was first translated to English through Google Translate if it was a non-English speaking country. The webpage was navigated manually, and search terms were used to ascertain nutrition information. The search terms included: nutrition, food, meal, diet, carbohydrate, protein, fat, hydration, supplements and vitamins and were typed in both in English and the language of the webpage. If items came up that were unable to be translated, these were excluded from the synthesis. The search terms were discussed amongst the review team and deliberately chosen for simplicity, hoping to replicate what a coach, gymnast or parent might look for. If an NGB's webpage could not be found, this was recorded as information inaccessible. If an NGB's webpage was found but there was no information accessible regarding nutrition, these were still included in the synthesis. Two researchers completed the search to minimise the risk of missing data and were cross-checked to ensure the data was aligned.

## Results

Fifty-two governing bodies were included in the search, 48 of which we could access a website and 15 had information regarding nutrition (Table 1). Table 2 provides a summary of the nutrition related information on each website. The most frequent types of content were macronutrients (8), energy intake/expenditure/balance/availability (6), supplements (5), growth and maturation, hydration, micronutrients, RED-S (4), weight management, more general dietary advice (3), training/recovery/competition (2), alcohol and injury (1). The information was portrayed via a webinar/seminar/course, recommended external links (6), infographic/poster (4), booklet, anecdote and recipes (all 2). Most of the information was not targeted at a specific audience, but some NGBs had resources directed at coaches (6), athletes (3) and parents (2).

**Table 1.** A list of national governing bodies and the availability of information.

Federation Members Providing nutrition or related support on their web pages	Federation members with accessible web pages but no nutrition information	Federation members with no webpage found
Australia	Austria	Armenia
Brazil	Albania	Bulgaria
Canada	Azerbaijan	China
Denmark	Belarus	India
Estonia	Chinese Taipei	
Finland	Columbia	
Germany	Croatia	
Great Britain and Northern Ireland	Cyprus	
Greece	Czech Republic	
Italy	Egypt	
Japan	Georgia	
Netherlands	Hongkong	
Norway	Hungary	
Romania	Ireland	
United States of America	Israel	
	Jordan	
	Kazakhstan	
	Korea	
	Latvia	
	Lithuania	
	Malaysia	
	Malta	
	New Zealand	
	Poland	
	Russia	
	Slovakia	
	Slovenia	
	South Africa	
	Switzerland	
	Turkey	
	Ukraine	
	Uzbekistan	
	Vietnam	

**Table 2.** A summary of information on each National Governing Body website.

NGB	Information
*Australian Gymnastics (Gymnastics Australia)*	Australian Gymnastics provided a booklet on body positive guidelines comprising of detailed recommendations. These included keeping conversations about food to a minimum, disseminating nutrition information must be evidence based, nutrition education should be delivered by dieticians only, weight should only be taken for body composition tracking purposes and the information should be kept private. Also available was a document titled ‘Disordered Eating Prevention and Early Intervention Policy’ which highlighted potential risk factors of disordered eating. A document titled ‘Guiding Values for Creating a Body Care Culture’ which focussed on the importance of eating enough energy to support daily tasks, avoiding giving food unhelpful labels and avoiding choosing foods based on emotions. The document also pointed to other foundations and networks of support that are available. The overall nutrition themes mentioned were weight management, macronutrients, supplements and health concerns such as Relative Energy Deficiency Syndrome (RED-S). The information was presented in a booklet and no specific target audience was identified. They suggested gymnasts sought advice from nutritionists, other sports personnel and medical professionals.
*Brazilian Gymnastics (Confederação Brasileira De Ginástica)*	A webpage was found with links to webinars with nutritionists and a webinar series. There was an anecdote titled ‘Part of the victory is won at the table’ where it was briefly mentioned that nutrition is an important part of performance. The information written on the website was brief and the webinars were in Portuguese so no nutrition themes could be extracted. The information was presented via anecdotes, external links and webinars and no specific target audience was identified. They suggested gymnasts sought advice from webinars and nutritionists.
*British Gymnastics (British Gymnastics)*	British Gymnastics (BG) showed a booklet on key recommendations and guidance when weighing gymnasts in which they clarified gymnasts should only be weighed for specific reasons such as personal preference, performance purposes as in the case of calculating power to weight ratio or countermovement jump height, or by a registered nutritionist as part of measuring body composition. There were also seven specific considerations about weighing gymnasts including; differences between body weight and composition, body weight fluctuations, the role of puberty in weight changes, weight in relation to injury, and power to weight ratio. BG provided a document titled Hydration Recommendations which provided information about checking hydration, replacing fluids after training, normalising drinking during training, refuelling and using the toilet during training. An infographic also presented the same information visually. They provided a document titled Health and Safety Guidance for Coaches in which there was a section titled Nutrition, highlighting that coaches should encourage a healthy balanced diet including carbohydrates, protein and fat. It emphasised that weight can fluctuate for gymnasts based on age and growth. Furthermore, attention was given to coach behaviour and conduct around weight loss, highlighting the risks associated with comments that could potentially damage gymnasts. BG provided anecdotes from gymnasts describing what they eat before performance or training. BG has partnerships with The Turmeric Company and Jaffa. The overall nutrition themes mentioned were weight management, hydration, macronutrients, growth and maturation as well as health concerns such as RED-S. The information was presented as an infographic poster, a booklet and anecdotes with some of the information specifically targeted at coaches. They suggested gymnasts seek advice from nutritionists, medical professionals and courses.
*Canadian Gymnastics (Gymnastics Canada)*	In the coach resources section of the website, there was a link titled ‘Sport Nutrition for Athletes and Coaches’. This provided a link to a page titled ‘Coaches Kitchen’ which showed a short online nutrition course and three pages worth of recipe ideas. The overall nutrition themes mentioned were macronutrients, recovery, dietary recommendations, energy intake, energy expenditure and supplements. The information was presented as an infographic poster, external link, recipes and courses with some information targeted at the athletes. They suggested gymnasts sought advice from nutritionists, other sports personnel and courses.
*Danish Gymnastics (Danmarks Gymnastik Forbund)*	Danish Gymnastics, provided links to some FIG training seminars from 2019 accessible on YouTube. They provided a link to a sports nutrition course to increase knowledge of the importance of dietary and fluid intake. The overall nutrition themes were not clear and the information was presented in the form of a webinar. Some of the information was targeted at the coach but no recommendations about seeking nutrition advice was provided.
*Estonian Gymnastics (Eesti Võimlemisliit)*	Estonia provide a link to a sports nutrition consultant for support. A separate website linked, displayed advice on how to support positive mental health and a section devoted to encouraging a balanced diet. There are conversation cards, tips to help positive eating and videos about a balanced diet. The overall nutrition themes were guidance on energy availability, energy balance, energy intake and energy expenditure. The information was presented as an infographic poster and no specific target audience was identified. They suggested gymnasts sought advice from nutritionists and other videos.
*Finland Gymnastics (Suomen Voimistelulitto)*	Finland provided several articles discussing different nutritional topics. The first article discussed general nutrition and the importance of eating to prevent energy deficit. The article highlighted the importance of losing weight slowly and in healthy way. It provided suggestions as to what should be on individual's plates. The second article focussed on snacks. The article discussed why carbohydrates are important for the brain and to maintain energy, different types of proteins, carbohydrate and vegetable options, and a recipe for mango, coconut and chia pudding. The article instructed on reading food labels, increasing awareness of what different foods do and advocates a plant based diet. The third article was titled ‘How should a gymnast eat?’. The article had several subheadings; ‘Food peace guaranteed’ where energy requirements and individual differences are highlighted, ‘The relationship with food is born in childhood’ where information discussing the importance of childhood habits, food being about well-being and avoiding reward and punishment practices with food are discussed, and ‘We have to talk about food’ where emphasised the importance nutrition being a subject not to be avoided and encourage coaches to talk about it. The overall nutrition themes were guidance on macronutrients, energy availability, energy balance, energy intake and energy expenditure. The information was presented on the website as articles and some of the information was targeted at athletes. No recommendations about seeking nutrition advice were provided.
*German Gymnastics (Deutscher Turner-Bund)*	Germany provided two articles. The first article discussed information regarding nutrition and the role of different macronutrients. The second article was titled ‘Power is Edible’ where a nutritionist answered questions relating different nutrition topics primarily focussing on macronutrients. There is a webpage that discusses hydration strategies and provides four top tips to stay hydrated. Other articles provided information on nutrition to maximise health and provide additional links to the German Nutrition Society. The overall nutrition themes were guidance on macronutrients, hydration, micronutrients, dietary recommendations, growth and maturation as well as supplements. The information was presented as external links and no specific target audience was identified. No recommendations about seeking nutrition advice were provided.
*Greek Gymnastics (Γυμναστική Ομοσπονδία Ελλάδος)*	Greece provided a nutrition webinar titled ‘Returning to training with proper nutrition’ which was given by a dietician-nutritionist. Also advertised was a webinar about nutrition during the COVID-19 pandemic. The overall nutrition themes were not clear and the information was presented in the form of a webinar. No specific target audience was identified, and no recommendations about seeking nutrition advice were provided.
*Italian Gymnastics (Federazione Ginnastica d’Italia)*	There were YouTube clips from a Symposium considering injury prevention and nutrition. There were also a variety of articles including some summarising information about preventing eating disorders and highlighting signs and symptoms, and one about training fasted. The overall nutrition themes were not clear and the information was presented in the form of a webinar and external links. No specific target audience was identified and gymnasts were recommended to seek advice from webinars and seminars.
*Japanese Gymnastics (日本体操連盟)*	Japanese gymnastics offered two textbooks available for purchase which included a chapter on nutrition for Rhythmic Gymnastics. This information was unavailable unless purchased. The overall nutrition themes were not clear and the information was presented in the form of a book aimed at coaches. No additional recommendations were made about seeking nutrition support.
*Norway Gymnastics (Norges Gymnastikk)*	Norwegian gymnastics advertised a food service of meals that could be delivered and eaten in the centre or taken back to their cabin. There is a link to a menu and which advertised that it could act as an efficient system of eating in amongst training or competitions. The overall nutrition themes were not clear as it was simply a food service menu on their website for athletes going on competitions.
*Romanian Gymnastics (Federaţia Romaână de Gimnastică)*	Romanian gymnastics displayed a nutrition education course, but there was no clear way of signing up for it. The overall nutrition themes were not clear as it was simply an advertised nutrition course with no specified target audience.
*The Dutch Gymnastics (Nederlandse Gymnastiek Federatie)*	The Netherlands provided access to a webpage that had 5 top tips aimed at parents for young athletes. There was also a separate link titled: ‘Protein Toppers, what are the most proteins in?’ This link proceeded to highlight different proteins and describe some the benefits, calorie content and other nutrient content of each. There are additional webpages that highlight the signs and symptoms of disordered eating. The overall nutrition themes were guidance on macronutrients, micronutrients, growth and maturation, energy availability, energy balance, energy intake and energy expenditure. the information was presented in the form of an external link aimed at parents, guardians and coaches. Additional recommendations that were made directed individuals to complete a nutrition course.
*The United States of America (USA) Gymnastics (USA Gymnastics)*	USA Gymnastics provided extensive information regarding nutrition advice, suggestions and information regarding nutrition through a variety of infographic posters. These included information on; nutrition concerns for gymnasts, fuelling strategies for performance, fuelling strategies with regards to snacks, vegan and vegetarian nutrition, altitude, recovery, hydration, travel, nutrients for bone and joint recovery, nutrients for soft tissue injury. The overall nutrition themes were guidance on alcohol, hydration, macronutrients, micronutrients, recovery, training, competition, dietary recommendations, growth and maturation, energy availability, energy balance, energy intake and energy expenditure, travel, supplements, health concerns such as RED-S and injury. The information was presented in the form of infographic posters, external links, recipes and webinars. It was clear that the information was directed at coaches, athletes and parents or guardians. Additional support was recommended from research, medical professionals and webinars.
*International Federation of Gymnastics (FIG)*	The International Federation of Gymnastics (FIG) provided a variety of information topics in the form of a research paper. The headings included weight management, energy needs and expenditure, nutrition which proceeded to add more detail regarding specific nutrients, supplements and junk food. They provided a section on eating disorders emphasising the risk they have for both health and performance. Finally there is a section on recommendations for optimum weight management within the field of gymnastics The FIG also have a coach education channel on YouTube which is available to open subscription, one of which is a video titled ‘Improving performance with nutrition, sleep and rest’ which primarily focussed on nutrition for recovery. In the video, the speaker highlights the three Rs of recovery; Refuel: glycogen stores with carbohydrates, Repair: muscle damage with proteins and Rehydrate: with fluids and gives food examples throughout. The overall nutrition themes were guidance on weight management, hydration, macronutrients, micronutrients, supplements and health concerns such as RED-S. The information was presented as an article and some information was directed at coaches. No other recommendations were made to seek nutrition advice.

### Findings

The purpose of this study was to ascertain what nutrition advice is freely available to gymnasts, coaches and parents or guardians on a global platform using lay search terms. Although there was a variety of nutrition information found across different NGBs, it also appeared that free public nutrition advice is limited and sometimes conflicting across the global platforms.

Out of the eight NGBs to mention macronutrients, five different NGBs, including the FIG (America, Germany, United Kingdom, The Netherlands, FIG), made more detailed references, but only USA Gymnastics provided specific recommendations. The recommendations provided by USA Gymnastics were detailed and provided diagrams to make information clear. The information provided by USA Gymnastics was lower than literature recommendations for carbohydrates (by 2 g.kg.bw^−1^) and higher for protein (by 0.3 g.kg.bw^−1^) (Bonci, 2010; Cotugna et al., 2005; Dallas et al., 2017; Desbrow, 2021). The remaining NGBs, including the FIG, provided some detail about the function of macronutrients and why they are important, but they did not discuss any specific recommendations. For example, British Gymnastics encouraged athletes to consume ‘appropriate’ amounts of macronutrients, however, there was no information or advice as to what ‘appropriate’ amounts are.

Conflicting information, or at least information that would benefit from further clarity, was evident on some websites. This might, at times, be due to translation issues, but one example was Germany's NGB which provided two articles discussing nutrition one of which highlighted a food first approach, emphasising the importance of obtaining protein from natural sources, and then in brackets wrote ‘no shakes’. In another article titled ‘Power is Edible’, there is a comment from a nutritionist saying ‘I definitely believe in protein in the shake area’. Whilst this comment is also accompanied by discussions about the importance of this alongside a healthy diet, it could be open to misinterpretation. Furthermore, the Netherlands NGB provided some in-depth advice about different carbohydrates encouraging the consumption of mostly slow-releasing carbohydrates and avoiding simple carbohydrates. Where this is a widely accepted healthy option, it also omits information about the benefits provided by the FIG which says that quick releasing carbohydrates can be useful for recovery or quick bursts of energy (Binder, 2013).

Another area of potential conflict was around hydration. British Gymnastics provided an in-depth document describing the importance of hydration alongside an infographic to display the information visually. The USA also provided a detailed infographic with the addition of specific fluid recommendations which are concurrent with the America College of Sports Medicine guidelines (Thomas et al., 2016). However, the complication with these recommendations is they focus largely on body weight to determine the volume of fluid losses. The FIG actively discourages the regular weighing of gymnasts due to the stress it places on them and the reinforcing unhealthy narratives around body weight (Binder, 2013). Therefore, providing recommendations that require body weight monitoring might not be the most effective way to measure hydration in gymnasts.

A final example of potential conflict relates to the search function within the NGB websites. Most of those containing a search function searched only within the site itself. However, others had a different hosting platform that returned external hits at the top of the list (Brazil, Canada, Egypt) ahead of those within the site. These were often links to supplement companies, weight loss organisations or food boxes which may or may not include appropriate advice and guidance. This is also unhelpful as NGBs could be seen as recommending a variety of services some of which might not be appropriate for gymnasts.

There was a wide range of other information provided by different NGBs surrounding nutrition. Some information was detailed but did not appear specific to gymnastics (e.g., Finnish Gymnastics). Other information provided recipes and was more specific to coaches (e.g., Gymnastics Canada, BG). The information specifically targeted at coaches was not only detailed but also potentially useful for athletes and parents. However, as it was titled for coaches, it might mean that other parties would not look or read the information assuming it is not for them.

### Summary

In summary, some NGBs provided more information than others, with few providing specific guidance on nutrient intakes. This could leave readers feeling reassured about the role of certain nutrients but also unsure as to how much they should be eating of each macronutrient. Some organisations provided advice that would benefit from further clarity, or advice in conflict with the FIG recommendations, which might lead to confusion. Furthermore, guidance provided around hydration focussed on weight related guidelines, which could be difficult to enact, particularly with the concerns around overemphasis on weight within the gymnastics community. Therefore, to provide effective support for the gymnastics population, clear guidelines and support should be available to ensure optimal energy availability, health and performance.

### Limitations

The retrieved results are limited to the terms used to search the websites, and it is possible that information might have been missed. Therefore, whilst we might not have a definitive list of content, any content missed is likely difficult to locate by other website users too. Google Translate was used to interpret webpages and translate search terms meaning no reference point was given to confirm the validity of the webpages. Some NGBs provided PDF style documents that could not be easily translated, therefore, nutrition information might have been excluded due to translation barriers. Some NGBs might have included a member paywall which potentially prohibited information being sought. Finally, it should be acknowledged that some NGBs might keep their nutrition information private for competitive reasons.

### Recommendations

*Dietary recommendations:* A lot of general guidance was found among the NGB websites, with most seeming to stay away from providing specific dietary recommendations. More specific advice, as to how much of each nutrient should be consumed, would provide additional essential information to enable athletes to achieve optimal energy availability. Alternatively, NGBs could signpost their members to relevant professionals such as dieticians or nutritionists, for additional and specific support.

*Hydration:* Hydration guidelines were only present from a couple of NGBs and some of those referred to guidelines relating to weight which could only be possible through regular weighing of gymnasts. This is actively discouraged by the FIG and other NGBs (USA, Australian and British Gymnastics) due to the risk of over-emphasising weight changes and fluctuations. Therefore, clarity around measuring hydration status and the provision of tools would benefit the gymnastics community.

*Centralised advice:* Some of the advice present among NGBs was conflicting or contradictory to the FIG recommendations. Therefore, the gymnastics community would benefit from a centralised nutrition platform approved by the FIG to give clear guidance to all athletes, coaches and parents.

*Website search hosting:* NGBs should be aware of any external links that feature at the top of search lists ahead of hits on their own website, as these may be accessed ahead of their own quality assured content.
